# Sudden Cardiac Arrest in a Pregnant Woman with Wolff–Parkinson–White Syndrome with Fractionated Delta Wave Due to Middle Cardiac Vein Diverticulum

**DOI:** 10.19102/icrm.2026.17051

**Published:** 2026-05-15

**Authors:** Rahel M. Sileshi, Amulya Gupta, Angela Martin, Siva Soma, Madhu Reddy, Amit Noheria

**Affiliations:** 1 Department of Cardiovascular Medicine, University of Kansas Medical Center, Kansas City, KS, USA; 2 Department of Obstetrics and Gynecology, University of Kansas Medical Center, Kansas City, KS, USA

**Keywords:** Fractionated delta wave, middle cardiac vein diverticulum, sudden cardiac arrest, Wolff–Parkinson–White

## Abstract

A 26-year-old woman at 34 weeks’ gestation presented with out-of-hospital cardiac arrest due to ventricular fibrillation. Her electrocardiogram (ECG) showed sinus rhythm with a short P–R interval and an unusually fractionated delta wave. The delta wave was positive in leads I and V2–V6, isoelectric in V1, and negative in inferior leads. An electrophysiology study revealed antegrade accessory pathway conduction with the earliest ventricular activation in the posteroseptal region. Three radiofrequency (RF) ablation procedures, including attempts with pulsed field ablation, were ultimately unsuccessful. Cardiac computed tomography angiography revealed a diverticulum of the middle cardiac vein (MCV). Irrigated RF ablation within the diverticulum successfully eliminated the accessory pathway conduction. We propose that multiple fibers of the coronary sinus or the MCV muscular coat may interface via the diverticulum with the ventricle, leading to multiple wavefronts of ventricular pre-excitation, and that the resultant delta-wave fractionation may allow identification of such an epicardial accessory pathway.

## Introduction

Wolff–Parkinson–White (WPW) syndrome is a rare cardiac conduction abnormality that is caused by an accessory atrioventricular (AV) pathway. Although the exact incidence of WPW syndrome in pregnancy is unknown, supraventricular tachycardias (SVTs) overall affect approximately 13–24 women per 1000 pregnancies.^1^ WPW syndrome can present with a range of tachyarrhythmias, from atrioventricular re-entrant tachycardia (AVRT) to pre-excited atrial fibrillation (AF). The most serious complication is sudden cardiac death (SCD), which occurs when pre-excited AF with a rapid ventricular response degenerates into ventricular fibrillation (VF), causing circulatory collapse. In this case report, we describe a previously asymptomatic pregnant woman who presented with sudden cardiac arrest due to WPW syndrome caused by an accessory pathway arising from a middle cardiac vein (MCV) diverticulum. We highlight the fractionation of the delta wave on electrocardiogram (ECG) as a potential clue for the “epicardial” coronary venous diverticulum-related accessory pathway.

## Case presentation

### Initial presentation

A 26-year-old G3P1A1 woman with no significant past medical history, at 34 weeks and 4 days of gestation, experienced sudden lightheadedness and chest pain while dining at a restaurant, followed by loss of consciousness. She received cardiopulmonary resuscitation in the field and had a return of spontaneous circulation after two external defibrillation shocks for VF. On arrival, her blood pressure was 159/108, pulse rate was 130 beats/min, and the 12-lead ECG showed sinus tachycardia with ventricular pre-excitation and occasional more pre-excited premature atrial complexes. 

**Figure 1A** shows a subsequent ECG tracing demonstrating sinus rhythm with a short P–R interval and a delta wave, positive in leads I and V2–V6, isoelectric in V1, and negative in the inferior leads, localizing the accessory pathway to the posteroseptal and possibly epicardial coronary venous region. Peculiarly, the delta wave demonstrated unusual fractionation, especially in the inferior leads.

**Figure 1:**
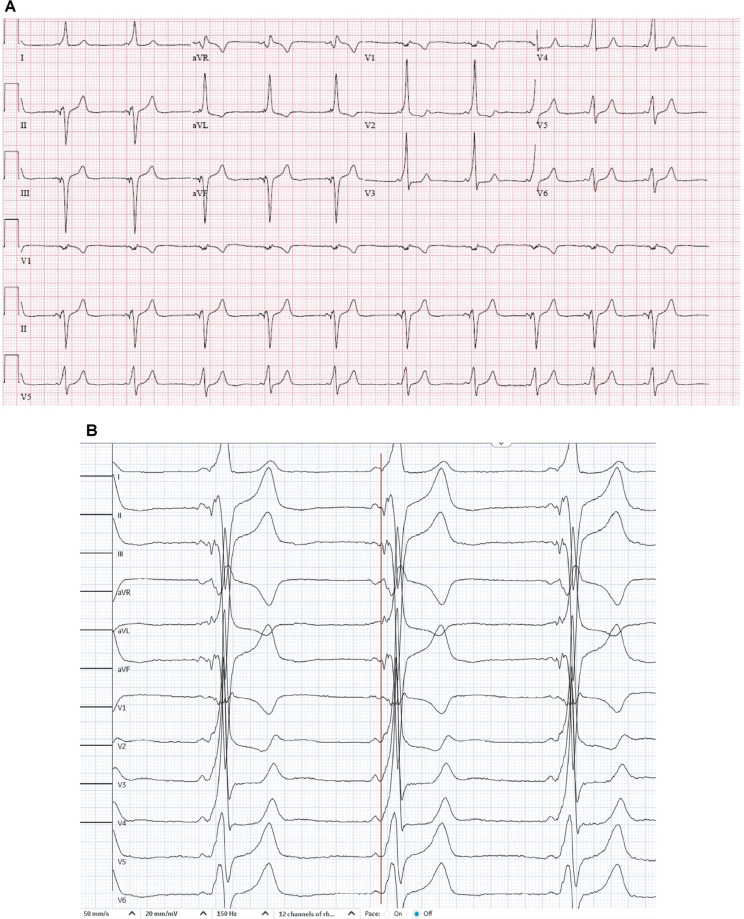
**A:** Baseline 12-lead electrocardiogram showing Wolff–Parkinson–White pattern. **B:** A gained up (20 mm/mV) and stretched (50 mm/s) synchronous 12-lead electrocardiogram layout of the electrocardiogram, with the beginning of the delta wave marked by the vertical line.

A bedside obstetric ultrasound revealed a viable fetus estimated at 35 weeks with biometry. Due to the late preterm gestational age with cardiac arrest of unknown etiology at the time, a decision was made to proceed with emergency cesarean delivery. Transthoracic echocardiogram showed a dilated left ventricle (LV) with an LV end-diastolic volume index of 103 mL/m^2^, normal wall thickness, and a reduced ejection fraction of 40%. The electrophysiology (EP) team was consulted and recommended an EP study and catheter ablation for definitive management.

### First electrophysiology study

On her second day of admission, the patient underwent an initial EP study under general anesthesia. Anterograde conduction via a posteroseptal accessory pathway with an effective refractory period of <200 ms was observed. The patient converted to a mid-RP tachycardia (likely orthodromic re-entrant tachycardia) during the initial phase of the EP study, with a cycle length of 250 ms, which spontaneously terminated. She also experienced a brief episode of AF that resolved spontaneously. Mapping with atrial and ventricular pacing localized the earliest signals to the posteroseptal region of the tricuspid annulus and the coronary sinus (CS) ostium. Irrigated radiofrequency (RF) lesions at 35 W were delivered at the right posteroseptum and the CS ostium without affecting the accessory pathway. After transseptal left atrial access, the earliest antegrade ventricular activation during atrial pacing with AV fusion was mapped to the posteroseptal region abutting the prior right posteroseptal lesions. To ensure no fusion between AV nodal conduction and accessory pathway conduction, adenosine (12 mg) was administered while mapping. The RF ablation lasted for 45 min but only transiently suppressed accessory pathway conduction. Following unsuccessful ablation of the accessory pathway, the patient was scheduled for a repeat EP study, with consideration of performing CS venography and pulsed field ablation (PFA). Cardiac magnetic resonance imaging (MRI) performed 3 days after the attempted ablation showed diffuse hypokinesis with grade III LV diastolic dysfunction but no evidence of edema/inflammation or late gadolinium enhancement.

### Second electrophysiology study

A second EP study was performed 7 days after her initial presentation under monitored anesthesia care. Retrograde atrial activation was mapped to the left posteroseptal region, but irrigated RF ablation at 40 W failed to eliminate the accessory pathway. Subsequent ablation at the right posteroseptal region at 40 W successfully eliminated accessory pathway conduction. The patient was discharged in normal sinus rhythm, with no evidence of pre-excitation on ECG. A 30-day ambulatory cardiac monitor later demonstrated recurrence of accessory pathway conduction 1 week after discharge. She also completed an exercise treadmill test. Her peak heart rate was 181 beats/min, and she had pre-excitation during the stress test.

### Third electrophysiology study

The patient underwent a third catheter ablation procedure 2.5 months after her initial presentation, performed under nurse-administered moderate sedation. Intracardiac echocardiography (ICE) identified a circular structure near the earliest activation site consistent with the MCV. The ablation catheter was advanced to this region, and both the proximal CS and MCV were mapped. Irrigated RF ablation lesions were delivered along the right ventricular (RV) posterior septal annulus, basal RV posterior septum (using a curling catheter to reach around the tricuspid leaflet), proximal MCV, and left-sided posterior septal annulus. The earliest retrograde atrial signals persisted on the left posterior/posterolateral region.

The PFA was then attempted using a Farawave™ (Boston Scientific, Marlborough, MA, USA) 31-mm pentaspline catheter at the right posteroseptal tricuspid annulus. A basket configuration with a catheter in the right atrium, pointing toward the posteroseptum, was employed, as the flower configuration was too large for precise positioning and to avoid injury to the AV node. Accessory pathway conduction was eliminated after multiple applications. There was a transient AV block during one PFA delivery. However, accessory pathway recurrence was noted 4 h post-procedure. The decision was made to discharge the patient on 150 mg of flecainide administered orally twice daily to suppress the accessory pathway with a plan for a repeat ablation. An ECG obtained 4 days later showed suppression of accessory pathway conduction with flecainide and an intraventricular conduction delay with QRS duration of 124 ms. However, accessory pathway conduction recurred on subsequent ECGs **(Figure 2)**. Later, computed tomography angiography (CTA) confirmed the presence of a coronary venous diverticulum **(Figure 3)**. Repeat cardiac MRI showed normalization of LV ejection fraction (53%) and an LV end-diastolic volume index of 75 mL/m^2^ without evidence of inflammation or fibrosis, except for a localized region of delayed gadolinium hyperenhancement in the basal inferoseptal subendocardial region related to prior ablation.

**Figure 2:**
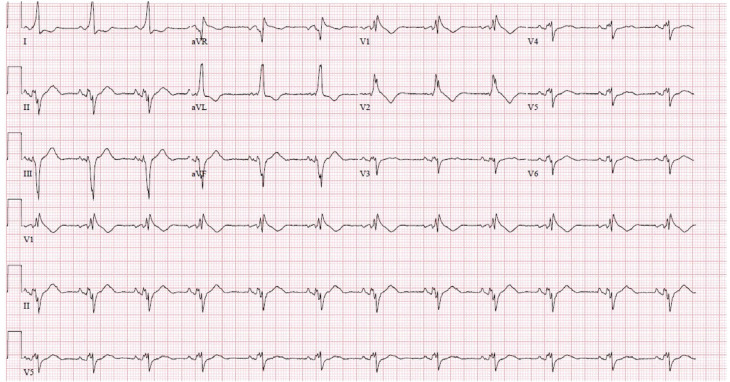
Electrocardiogram with recurrent Wolff–Parkinson–White pattern despite flecainide following the third ablation procedure. The wider QRS complex with a different morphology compared with the initial electrocardiogram is probably a combination of different degrees of fusion between the accessory pathway and atrioventricular node conduction at two different time points and QRS widening due to flecainide.

**Figure 3:**
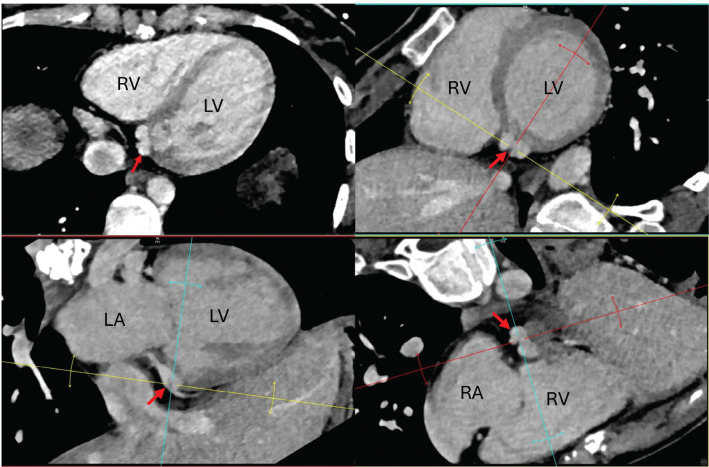
Computed tomography angiography of the coronary venous system demonstrating a diverticulum of the middle cardiac vein. Top left: axial slice with red arrow showing the middle cardiac vein diverticulum. The other three panels: Three-dimensional oblique cuts with crosshairs centered in the middle cardiac vein diverticulum. *Abbreviations:* LA, left atrium; LV, left ventricle; RV, right ventricle.

### Fourth electrophysiology study

The fourth ablation procedure was performed 4 months and 3 weeks after her initial presentation. The procedure was planned under general anesthesia with the possibility of percutaneously accessing the pericardial space for epicardial mapping/ablation. Bilateral femoral venous access was obtained. The Agilis™ sheath and ICE catheter were advanced through the right femoral vein, while the His and decapolar CS catheters were placed via the left femoral vein. ICE imaging from the right atrium identified the MCV diverticulum **(Figure 4)**. Through the Agilis™ sheath, a DECANAV™ (J&J Medtech, New Brunswick, NJ, USA) D-curve catheter was gently advanced into the MCV diverticulum. The ventricular electrogram within the MCV was 20 ms pre–delta wave. After the Agilis™ sheath was advanced into the ostium, the mapping catheter was withdrawn, and a balloon-tipped Arrow™ (Teleflex Inc., Wayne, PA, USA) catheter was advanced over a wire. Contrast injection was performed in both right and left anterior oblique projections to delineate the anatomy of the MCV diverticulum **(Figure 5)**. A ThermoCool® (J&J Medtech) Nav D–F curve catheter was subsequently advanced for detailed mapping. The earliest ventricular activation during sinus rhythm and atrial extra stimulation was localized to the RV posterior septum and the MCV diverticulum, with the earliest signal (30 ms pre–delta wave) recorded at the neck of the diverticulum **(Figure 6A)**. Sequential irrigated RF applications within the diverticulum and at its neck resulted in immediate elimination of pre-excitation **(Figure 6B)**. Consolidation lesions were applied circumferentially around the neck of the diverticulum, with power titrated up to 35 W.

**Figure 4:**
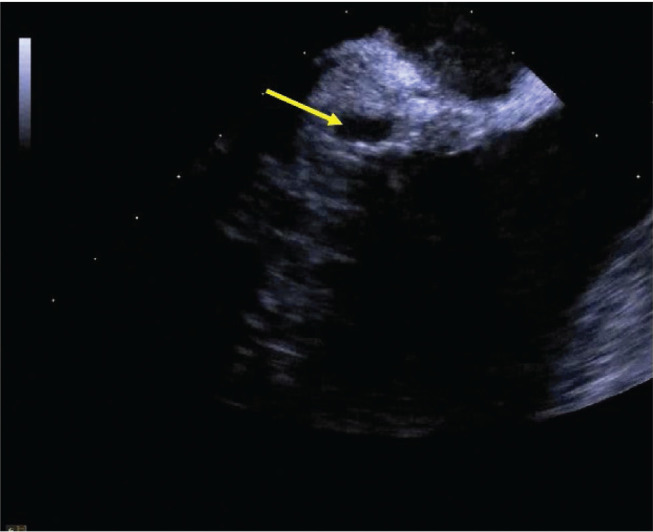
Intracardiac echocardiography of the middle cardiac vein diverticulum (arrows) imaged from the right atrium.

**Figure 5:**
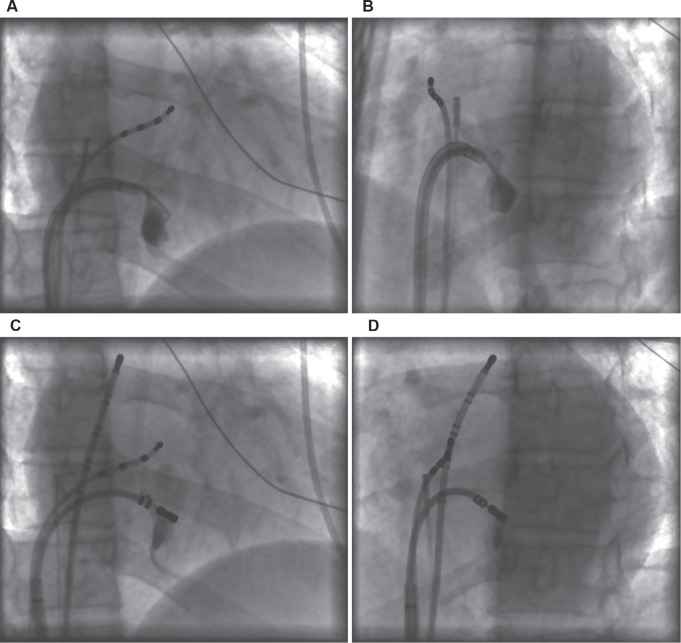
Fluoroscopic images demonstrating mapping and ablation of the middle cardiac vein diverticulum. Right **(A)** and **(B)** left anterior oblique views showing contrast injection into the middle cardiac vein diverticulum to delineate its anatomy before ablation. Right **(C)** and **(D)** left anterior oblique views showing ablation catheters positioned in the middle cardiac vein diverticulum.

**Figure 6:**
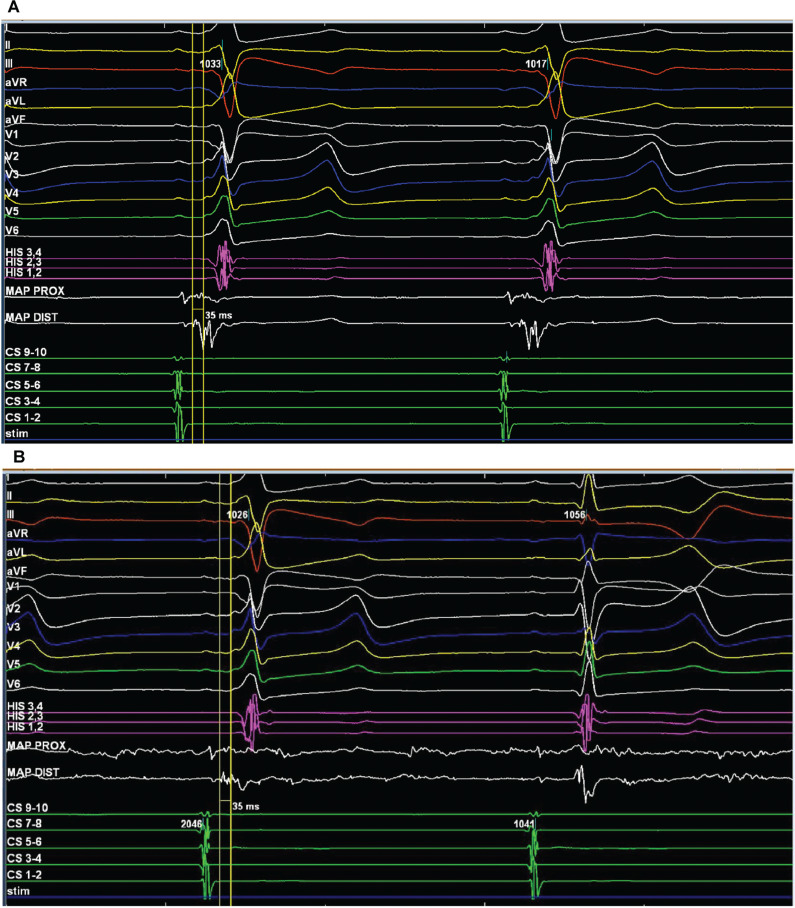
Intracardiac electrograms. The 12-lead electrocardiogram and recordings from the HIS, MAP, and CS are shown. **A:** Sharp “accessory pathway” potential 35-ms pre–delta wave recorded at the neck of the middle cardiac vein diverticulum. **B:** Elimination of pre-excitation with the delivery of radiofrequency ablation at the location of the accessory pathway potential.

Following ablation, a comprehensive EP study was performed. Intravenous adenosine (12 mg) and isoproterenol (10 μg/min) were administered. There was no evidence of retrograde ventriculoatrial conduction at a cycle length of 600 ms, and SVT was non-inducible. No accessory pathway conduction recurred during 30 min of observation or with adenosine/isoproterenol testing. Dual AV nodal physiology was noted during isoproterenol infusion, but no echo beats or inducible atrioventricular nodal re-entrant tachycardia were observed. Para-Hisian pacing after ablation demonstrated a nodal response. Flecainide was discontinued following successful ablation. A post-ablation ECG is shown in **Figure 7**. The patient has remained asymptomatic without palpitations or tachyarrhythmia since her initial presentation, without any evidence of WPW on the 3-month follow-up ECG. **Table 1** summarizes the procedural details and outcomes of four ablation attempts.

**Figure 7:**
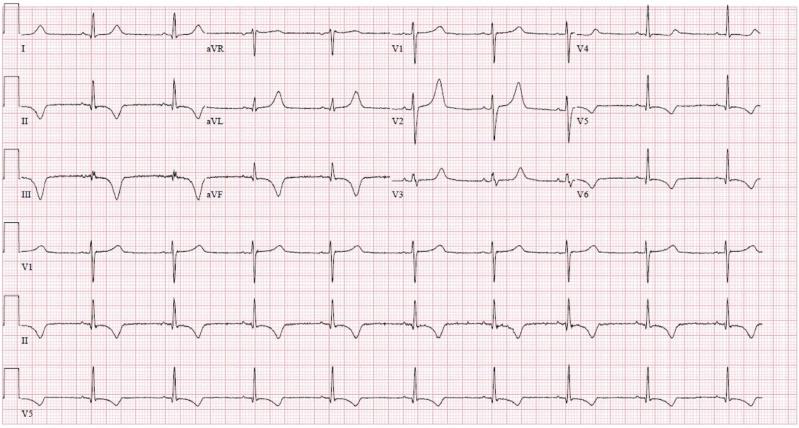
Post-ablation electrocardiogram showing resolution of Wolff–Parkinson–White pattern. Note abnormal repolarization due to T-wave memory from previously pre-excited QRS.

**Table 1:** Procedural Details and Outcomes of the Accessory Pathway Ablations

Procedure	Time After Presentation	Ablation Energy	Targeted Location(s)	Accessory Pathway Outcome
First	1 day	35 W	– Right posteroseptum annulus – CS ostium – Left posteroseptum annulus	Conduction persisted
Second	7 days	40 W	CS ostium	– Conduction acutely eliminated – Antegrade conduction recurred 1 week later
Third	2 months, 2 weeks	Up to 50 W	– Annular RV posteroseptum – CS ostium/proximal MCV – Left posteroseptum	– Conduction acutely eliminated with PFA – Antegrade conduction recurred 4 h post-procedure – Flecainide started
		PFA (Farapulse)	Right posteroseptum annulus	
Fourth	4 months, 3 weeks	Up to 35 W	MCV diverticulum and its neck	Successfully eliminated

*Abbreviations:* CS, coronary sinus; MCV, middle cardiac vein; PFA, pulsed field ablation; RV, right ventricle.

## Discussion

The MCV, also known as the inferior or posterior interventricular vein, originates near the apex of the LV, runs in the posterior interventricular groove, and drains into the CS.^2,3^ The CS has a muscular coat that interfaces with the left and right atria and may extend into the MCV and the posterolateral vein (PLV). This extension of the CS muscular coat can also interface with the ventricular myocardium, with or without a CS, MCV, or PLV diverticulum, and result in WPW syndrome.^4^ Posteroseptal accessory pathways account for approximately 34.5% of all WPW pathways, and nearly one-third of these are epicardial connections that travel within the CS and its tributaries.^4–6^ Epicardial pathways arising from the CS diverticula, in particular, have shorter refractory periods that predispose patients to rapid ventricular rates during episodes of AF.^7^ To the best of our knowledge, this is the second case of sudden cardiac arrest as the initial manifestation of an epicardial accessory pathway reported in the literature.^8^ Additionally, our patient had her first manifestation of WPW, ie, sudden cardiac arrest, during the third trimester of pregnancy.

The risk of SCD in asymptomatic individuals with a WPW pattern is not precisely defined but is estimated at approximately 1.3 per 1000 person-years.^9^ Several features have been identified as predictors of SCD among asymptomatic patients with a WPW pattern, including an effective refractory period of the accessory pathway ≤240 ms, a shorter pre-excitation R–R interval during AF (≤250 ms), male sex, younger age, syncope, and induction of AVRT during EP study.^10^ However, electrophysiologic characteristics of the accessory pathway correlate poorly with the overall clinical severity of WPW syndrome; in one study, one-third of WPW cases with a life-threatening arrhythmic event would have been considered low-risk based on these EP study criteria.^11^ Nevertheless, due to the risk of SCD, WPW ablation should be considered in asymptomatic patients with high-risk features or multiple accessory pathways.^12^

The mechanism of cardiac arrest in our patient was inferred to be pre-excited AF, resulting in VF. Patients with WPW are at an elevated risk of AF even at younger ages. Even though other mechanisms, such as intrinsic atriopathy or ectopic activity from the accessory pathway itself, have been postulated, degeneration of AVRT into AF is probably the most likely cause.^13^ Pregnancy is a known trigger for SVT as well as AF, especially in the second and third trimesters.^14^ This is probably related to cardiac stretch, driven by hormonal changes in a volume-expanded state and increased adrenergic tone.^15^ Furthermore, peak resting sinus heart rate occurs around 34 weeks of pregnancy.^16^ Our patient experienced her cardiac arrest at 34 weeks of gestation.

Accessory pathways can be localized on the ECG using published algorithms.^17–19^ Epicardial accessory pathways associated with CS, MCV, or PLV diverticula may exhibit characteristic ECG findings. Specifically, a clearly negative delta wave in lead II, without an initial isoelectric component, is considered a relatively specific finding for these accessory pathways. In one center, this ECG finding identified an epicardial accessory pathway in 14 patients with prior failed posteroseptal ablation.^17^ In our case, the delta wave is unusual in that it has multiple fractionated components, including an initial sharp negative deflection in leads II, III, and aVF **(Figure 1B)**. We hypothesize that the multiple components may correspond to multiple muscular attachments from the MCV diverticulum to the basal inferior ventricular epicardium, generating multiple pre-excited wavefronts, as shown in **Figure 8**. We reviewed seven ECGs from published case reports of coronary venous diverticular accessory pathways. Five of them did not show such fractionation,^20–26^ while the remaining two appear to have fractionation in lead II.^22,23^ It is unclear how frequently such a pattern of delta-wave fractionation is observed.

**Figure 8:**
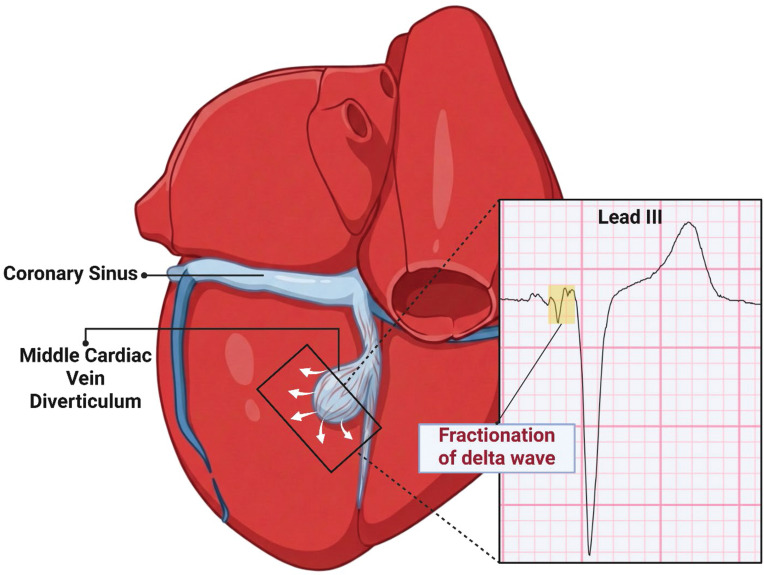
Schematic illustration of an epicardial accessory pathway within a middle cardiac vein diverticulum and its electrocardiogram manifestation. Muscular fiber attachments from the diverticulum to the basal inferior ventricular epicardium give rise to multiple pre-excited wavefronts, which are thought to underlie the delta-wave fractionation observed on electocardiography. *Note:* This figure is adapted from content available under the Creative Commons Attribution–ShareAlike 2.1 Japan license (https://creativecommons.org/licenses/by-sa/2.1/jp/deed.en; accessed January 15, 2025) and was changed into a cartoon-style illustration using BioRender.com, with the diverticulum and muscular fibers added using BioRender AI.

Epicardial accessory pathways are challenging to map and localize because of extensive CS–atrial connections, the oblique orientation of CS myocardial fibers, and the absence of a distinct ventricular insertion site.^6^ Additionally, the proximity of posteroseptal accessory pathways to the triangle of Koch, which contains the AV node and the coronary artery, further complicates ablation.^27,28^ Precise localization of the coronary venous anatomy using CTA and contrast venography can improve procedural planning.^26,29^ CS diverticula and surrounding cardiac structures can also be visualized using cardiac MRI. However, cardiac computed tomography provides better spatial resolution than MRI.^30^ Furthermore, atrial and ventricular pacing during an EP study can be used to assess the number and location of the accessory pathways.

RF ablation remains the standard treatment for accessory pathways, but its efficacy at epicardial sites is often limited by reduced lesion penetration and an increased risk of complications.^31^ The RF ablation in the venous system is also challenging because of impedance rise and the risk of charring and steam pops. Once the location is confirmed, the desired thermal energy can be delivered gently to eliminate the accessory pathway.^28^ At the time of the procedure, we only had access to the Farawave™ pentaspline catheter for PFA delivery, but a steerable focal ablation PFA catheter would be more advisable for targeting accessory pathways. Although PFA did not irreversibly eliminate conduction through this epicardial accessory pathway, a few case reports and series have demonstrated the feasibility and acute success of PFA for targeting accessory pathways, including posteroseptal locations.^32–34^

Finally, the management of WPW syndrome during pregnancy requires careful consideration to safeguard both maternal and fetal health. Catheter ablation is the definitive therapy, but it is usually deferred because of concerns of fetal radiation exposure. Although some studies have reported procedural success with appropriate abdominal shielding and zero-fluoroscopy techniques (using electroanatomic mapping and intracardiac echocardiographic guidance), the decision to ablate the accessory pathway in a pregnant woman must balance maternal arrhythmia burden and potential fetal complications, both from uncontrolled maternal tachycardia and from any potential radiation exposure.^35^ In most cases, antiarrhythmic agents such as flecainide can be used safely during pregnancy and provide adequate symptom control.^35^

## Conclusion

WPW syndrome is a rare cause of SCD in the absence of structural heart disease. It can be cured with accessory pathway ablation without the need for an implantable cardioverter-defibrillator for secondary prevention. Mapping of coronary venous accessory pathways can be technically challenging and requires careful elucidation within the coronary veins/diverticula. Electrophysiologists should have a high index of suspicion for an epicardial pathway using a coronary venous branch or diverticulum when a posteroseptal WPW presents with a negative delta wave in lead II with unusual features such as delta-wave fractionation, or if it is unusually hard to ablate or recurs after initial successful ablation.
